# Comparison of long-term effects of exergaming (Xbox one kinet) and companionship programs on attitude towards dementia and the older adults among adolescents: a quasi-experimental longitudinal study

**DOI:** 10.1186/s12877-022-03137-w

**Published:** 2022-05-19

**Authors:** Yuan-Ju Liao, Li-Chan Lin, Shiao-Chi Wu, Jung-Ling Fuh, I-Tsun Chiang, Bih-Shya Gau

**Affiliations:** 1 Department of Nursing, National Yang Ming Chiao Tung University, No.155, Section 2, Li-Nong Street, Beitou District Taipei, Taiwan; 2 Department of Nursing, Asia University, No. 500, Lioufeng Road, Wufeng District Taichung, 41354 Taiwan; 3 Institute of Health and Welfare Policy, National Yang Ming Chiao Tung University, No.155, Section 2, Li-Nong Street, Beitou District Taipei, Taiwan; 4 Faculty of Medicine, Schools of Medicine, National Yang Ming Chiao Tung University School, No.155, Section 2, Li-Nong Street, Beitou District Taipei, Taiwan; 5 Division of General Neurology, Neurological Institute, Taipei Veterans General Hospital, No. 201, Section 2, Shi-Pai Road, Taipei, Taiwan; 6 Department of Special Education, National Taiwan Normal University, No. 162, Section 1, Heping East Road., Da’ an District Taipei, Taiwan; 7 School of Nursing, College of Medicine, National Taiwan University, No. 1, Jen-Ai Road, Section 1, Zhongzheng District Taipei, Taiwan

**Keywords:** Older adult, Dementia, Adolescent, Attitude, Intergenerational programs

## Abstract

**Background:**

Many studies have been performed on the use of intergenerational programs to improve the negative attitudes and misunderstandings of adolescents toward older people with dementia. However, the findings of these studies are inconclusive. The aim of this study was to compare the long-term effects of exergaming (Kinect) and companionship programs on attitudes toward dementia and the elderly among adolescents.

**Methods:**

A quasi-experimental longitudinal design was used. A total of 200 adolescents aged 12–18 years old were recruited from nine schools in northern Taiwan. The adolescents were assigned to five different groups, namely, a 5-week exergaming group, a 5-week companion group, an 8-week exergaming group, an 8-week companion group, and a control group, using a single blinding procedure. Data collection was performed pretest, post-test and at 1, 3 and 6 months after the post-test. The long-term effects of the two programs (i.e., exergaming and companionship) were analyzed using a generalized estimating equation.

**Results:**

Regarding attitudes toward dementia, the 8-week exergaming group had a significantly better attitude than the control group at the 6-month follow-up (*p* < 0.001). Similarly, the results of the 8-week companion group also showed a significantly improved attitude compared with the control group at the 6-month follow-up (*p* = 0.041). Regarding attitudes toward the elderly, the 8-week exergaming group had a significantly better attitude than the control group at the 6-month follow-up (*p* < 0.001). The 8-week companion group had a similar effect on better attitude compared with the control group at the 6-month follow-up (*p* = 0.016). Furthermore, the 5-week companion group showed a significant improvement compared with the control group at the 6-month follow-up (*p* = 0.004).

**Conclusions:**

Spending companionship time with older adults is beneficial for improving the attitudes of adolescents toward the elderly. Furthermore, exergaming improves the attitudes of adolescents toward both dementia and older adults.

**Trial registration:**

Chinese Clinical Trial Registry: ChiCTR2100053003. Retrospectively registered on 07/11/2021.

## Background

The number of people with dementia is on the rise [1]. Ageism toward elderly individuals with dementia might lead to a higher risk in the current aging world and is a public concern [2]. Stereotyping processes begin at a young age [3], and adolescents have indirect contact with dementia through adverts and television [4]. Moreover, dementia myths exist among both adults and young people, such as dementia being a normal part of aging [5, 6] and a contagious mental illness [6]. The myths of dementia may have negative effects on attitudes toward older adults with dementia and on the resource allocation of dementia and family relationships. Today’s adolescents may well become the caregivers of tomorrow, and an increasing number of adolescents will interact with a grandparent [7] or parents [8] with dementia; thus, improving their attitudes toward older adults with dementia is crucial [9]. Previous studies have revealed that young cohorts exchange their perceptions [10, 11], attitudes toward [12, 13], and images of aging [14] through in-person contact with older adults. Studies have even shown that watching an intergenerational contact video improves adolescents’ attitudes toward older adults and aging knowledge [15, 16]. These types of results suggest that more frequent contact between adolescents and older adults is beneficial. In contrast, several studies have shown that interactions between adolescents and grandparents diagnosed with dementia had avoidance motives and negative emotions [17]. The intergenerational program revealed no significant improvement in attitudes toward dementia and older adults [18]. The above inconsistent research findings might result from failure to consider that optimal intergroup contact requires time to develop friendships and then reduce prejudice [19].

Exergaming is regarded as applicable based on the hypothesis of contact theory [20] that intergroup contact corresponding to lower intergroup prejudice is built into exergames. If those involved with intergroup contact have equal status, a common goal, and institutional support, position experience might counter prejudice and increase intergroup understanding. Exergaming can be defined as intermixing various physical exercises or physical full-body motions with video game play to achieve tasks or goals through visual perception, physical movement, and social interaction [21]. Research in the field of intergenerational exergaming showed that it created competition [22, 23] and collaborative elements [24–26] and can be used to encourage communication, collaboration, social interaction, friendship, and learning between healthy older adults and youth. Older adults, through intergenerational exergaming, have shown increased motivation, improved attitudes toward playing exergames with youth and lower levels of social anxiousness and loneliness [27], as well as improving adolescents’ attitudes toward older adults [13].

Although exergaming can provide a proper method of intergroup interactions, very few studies have suggested that exergaming among people with dementia and young people narrows the gap between generations or decreases the stigma of dementia [28]. Moreover, without considering the effect of time in intergroup contact, the findings of frequent contact on reverse perceptions and attitudes toward older adults by adolescents were inconclusive. Accordingly, the aim of the present study was to compare the long-term effects of combining exergaming and companionship programs on adolescent attitudes toward dementia and older adults with dementia.

## Methods

### Design

The study adopted a quasi-experimental longitudinal design. The adolescents were assigned to five groups: 5-week exergaming, 5-week companionship, 8-week exergaming, 8-week companionship, and a control group.

### Participants and settings

Adolescents were recruited from five junior high schools and four senior high schools in northern Taiwan from November 2016 to October 2018. The inclusion criteria were as follows: (1) 7^th^ to 12^th^ grade of study; (2) consent given by the school and parents to participate in off-campus services; and (3) the ability to communicate in Chinese or Taiwanese. The exclusion criteria were set as follows: (1) students who were orphaned; (2) consent not given by the school or parents to participate in off-campus services; and (3) the inability to communicate in Chinese or Taiwanese. The older adults were recruited from 15 daycare centers and had to meet the basic criteria of being 55 years old or older and having been diagnosed with mild to moderate dementia on their charts.

### Procedure

**Fig. 1:**
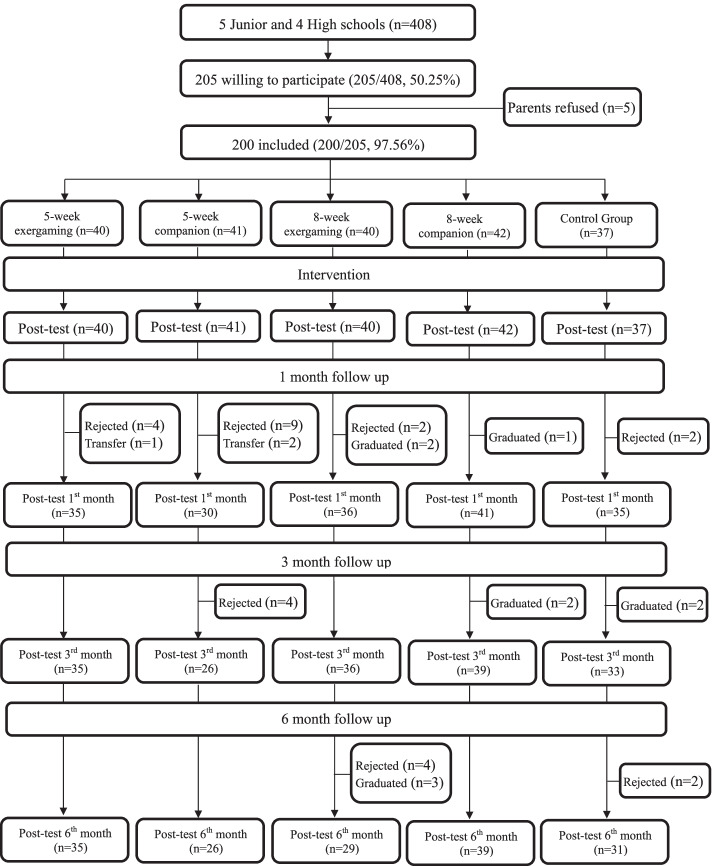
Flowchart showing overall research framework

After obtaining informed consent from the adolescents and their parents (or parent guardians), the older adults with dementia and their surrogates, data collection was performed prior to the intervention, after the intervention and at 1, 3 and 6 months after the post-test (see Fig. 1). The adolescents in the four experimental groups were assigned to 15 daycare centers close to their schools. Prior to the intervention, the adolescents were told only that they were going to serve the older adults, and therefore they did not know which experimental group they were assigned to. On entering their assigned daycare centers, the adolescents were primarily assigned one-on-one with older adult subjects based on their spoken languages and preferences. The number of adolescents exceeded that of the older adults, yielding 17 dyads of 1:2 adolescent and older adults for the 5-week exergaming and 8-week companionship groups and 15 dyads of 1:2 adolescent and older adults for the 5-week companionship and 8-week exergaming groups.

### Interventions

Prior to the intervention, the adolescents attended a one-hour training session in which they watched videos and slide shows about their assigned daycare centers and were briefed on the characteristics of the older adults in the center, the general physical and psychological characteristics of older adults with cognitive impairment, practical matters regarding the response of older adults with dementia, and general information about the intervention. Each intervention was structured as follows. (1) *Opening* (5 min): the adolescents introduced themselves to the older adults and explained the purpose of their visit and the planned schedule and content; (2) *Activity* (30 min): the adolescents led the older adults in performing warm-up exercises and then played the exergame or accompanied the older adults while performing a simple activity; and (3) *Ending* (5 min): the adolescents announced the end of the exergame or activity and said farewell.

### Intervention tool

For the 5-week and 8-week exergaming groups, the adolescents led the older adult participants in playing the chosen games for 40 min every week. The exergames were played on an Xbox One Kinect (Redmond, US). Three different games were used: *Bowling* (Clenched fists reach for the ball and throw the ball), *Target Shooting* (Face off directly against the opponent in split-screen cooperation, look for the crosshair symbol and shoot all targets) from the Kinect Sports Rivals game, and *Fruit Ninja2* (Slice fruit thrown into the air appears on the shadowy silhouette screen with movable arms and hands, each round only has a minute and thirty seconds) on the Xbox One. While playing the video games, both adolescents and older adults were required to engage in physical exercise to achieve the tasks through visual perception, physical motion, and cooperation.

For the 5-week and 8-week companionship groups, the adolescents accompanied the older adults as they performed regular daily activities in adult daycare centers (e.g., card games, singing, painting or whatever they chose) for 40 min each week.

The control group attended school activities without participating in the extracurricular intergenerational activity (Table 1).

**Table 1:** Content of intergenerational programs

Times/ Groups	8-week exergaming	8-week companion	5-week exergaming	5-week companion	Control
Before Intervention	Pre-intervention training (1 h)				School activities^a^
week1	Introduce and know each other				
	Bowling	Day care center regular activity	Bowling	Day care center regular activity	
week2					
week3					
week4	Bowling + Fruit Ninja		Bowling + Fruit Ninja		
week5					
week6	Fruit Ninja + Target Shooting		-	-	
week7					
week8					

^a^The control group attended school activities without participating in the extracurricular intergenerational activity

### Instruments

The Dementia Attitudes Scale developed by O'Connor and McFadden [29] and the Attitudes Toward Elderly modified scale developed by Hawkins [30] were short, concise, easy to understand, had modest psychometric properties, and took approximately 7–15 min to fill out. Both scales were used to evaluate the outcome of the intervention.

### Dementia attitudes scale

The attitudes of the adolescents toward dementia were measured using the Dementia Attitudes Scale developed by O'Connor and McFadden [29]. The scale comprises 20 items, such as “I feel confident around people with ADRD” and “I am comfortable touching people with ADRD”, and it is important to know the past history of people with ADRD. Each item is rated using a seven-point Likert scale ranging from 1 (strongly disagree) to 7 (strongly agree), yielding a total score ranging from 20–140. The higher the score, the better the attitude toward dementia. The scale comprises two dimensions: dementia knowledge and social comfort. Cronbach’s alpha value for the two dimensions ranged from 0.83 to 0.85. [29] In the present study, the scale was translated into Chinese and then confirmed via reverse translation by two bilingual researchers. Cronbach’s alpha of the pretest and post-test for the total scale ranged from 0.85–0.87, while Cronbach’s alpha of the pretest and post-test for the subscale of social comfort was 0.77 and the subscale of dementia knowledge ranged from 0.82–0.86.

### Attitudes toward the elderly

The Attitudes Toward Elderly scale was originally developed by Sanders et al. [31]. The modified scale by Hawkins [30] in 1996 was employed in the current study and comprises 20 questions that were evaluated using positive–negative adjective pairs (e.g., wise-foolish, happy-sad, independent-dependent). Each question was rated using a seven-point Likert scale ranging from 1 to 7, yielding a total score ranging from 20 to 140. Lower scores indicated a more positive attitude toward the older adults, and a score range of 70 to 90 indicated a neutral response. According to previous research, Cronbach’s alpha value for the scale was 0.97. In the present study, the original meaning of the scale was confirmed by reverse translation, and Cronbach’s alpha of both the pretest and post-test was 0.94.

### Background information form

The demographic characteristics of the adolescents were collected using a background information form and included their age, gender, school grade, parents' marital status, cohabitation status with grandparents, and previous history of participating in volunteer services for older adults.

### Data analysis

The differences between the experimental groups and control group were examined using descriptive statistics, Chi-square tests and one-way ANOVA tests. The long-term effects of the exergaming and companionship programs on the attitudes of the adolescents toward dementia and the older adults were analyzed using a generalized estimating equation. To avoid incurring the risk of Type II errors, no multiple comparisons correction was used with planned outcome analysis in this study [32].

## Results

**Fig. 2:**
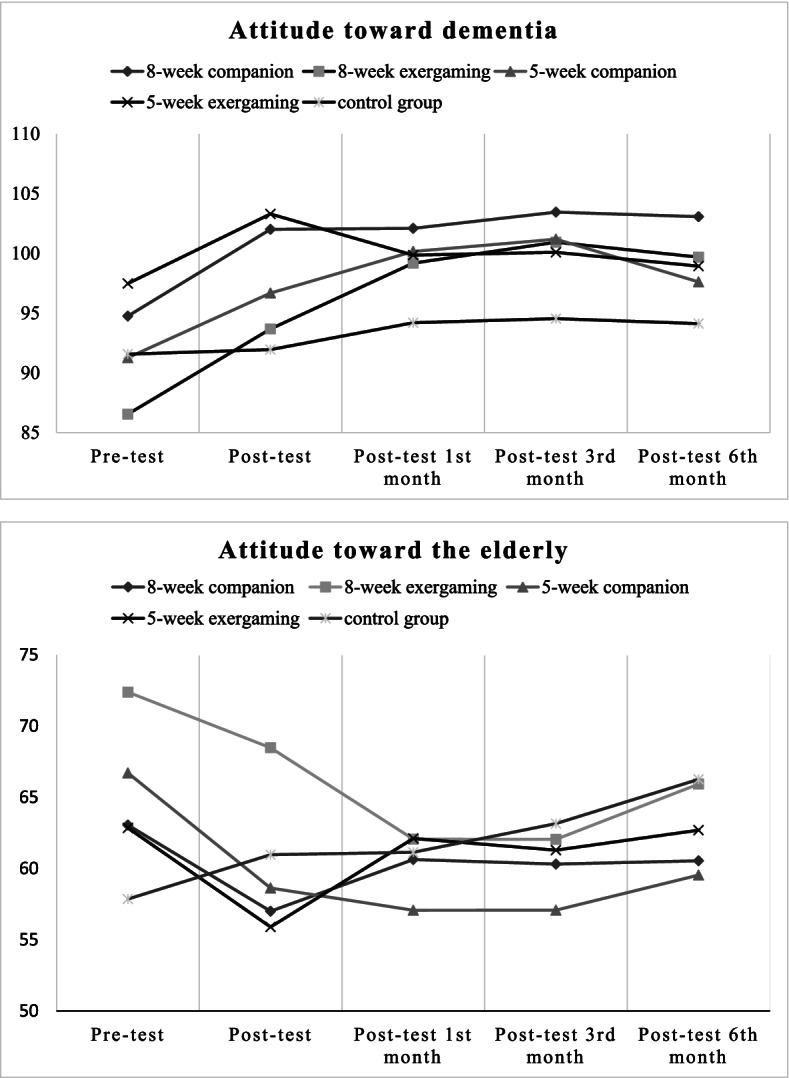
The mean score of attitudes toward the elderly and attitude toward dementia

Table 2 shows that the adolescents were mainly female and had an average age of 13.73 to 15.62 years. Significant differences existed among age, school grade and gender among the five groups (*p* < 0.001). In addition, more than 70% of the adolescents in the 5-week exergaming group and 8-week companion group had previous volunteer experience in serving older adults. Table 3 and Fig. 2 show the average attitudes of the adolescents in the five groups toward dementia and the older adults at different stages of the experiment (pretest, post-test and follow-up).

**Table 2:** Background information of adolescents

Items	EGI	EGII	EGIII	EGIV	CG	F / χ^2^	*p-value*	Post hoc Scheffe test
	(*n* = 40)	(*n* = 41)	(*n* = 40)	(*n* = 42)	(*n* = 37)			
	Mean (SD)/ n (%)	Mean (SD)/ n (%)	Mean (SD)/ n (%)	Mean (SD)/ n (%)	Mean (SD)/ n (%)			
Age (year)	13.73 (1.20)	14.73 (1.27)	15.00 (2.03)	15.62 (1.17)	14.16(1.37)	10.589	< .001***	CG < EGIV; EGI < EGII、 EGIII、EGIV
Gender						13.145	.011**	
Male	2 (5.0)	10 (24.4)	15 (37.5)	13 (31.0)	8(21.6)			
Female	38 (95.0)	31 (75.6)	25 (62.5)	29 (69.0)	29(78.4)			
School grade						34.426	< .001***	
7 to 9 grade	33 (82.5)	19 (46.3)	25 (62.5)	10 (23.8)	26 (70.3)			
10 to 12 grade	7 (17.5)	22 (53.7)	15 (37.5)	32 (76.2)	11 (29.7)			
Parents' marital status						12.417	.715	
Married	34 (85.0)	32 (78.0)	30 (73.2)	34 (82.8)	26 (70.3)			
Separated	5 (12.5)	8 (19.5)	7 (17.5)	7 (16.7)	8 (21.6)			
Windowed	1 (02.5)	1 (02.4)	3 (07.3)	1 (02.4)	3 (08.1)			
Living together with grandparents						2.541	.637	
Yes	14 (35.0)	8 (19.5)	12 (30.0)	12 (28.6)	10 (27.0)			
No	26 (65.0)	33 (80.5)	28 (70.0)	30 (71.4)	27 (73.0)			
Participation in volunteer services for the elderly						53.523	< .001***	
Yes	30 (75.0)	15 (36.6)	7 (17.5)	34 (81.0)	10 (27.0)			
No	10 (25.0)	26 (63.4)	33 (82.5)	8 (19.0)	27 (73.0)			

*SD* standard deviation, F-statistics are reported for one-way ANOVA, and chi-square statistic (χ^2^) was applied for Chi-square test; *** *p* < .001

EGI = 5-week exergaming group; EGII = 5-week companion group; EGIII = 8-week exergaming group; EGIV = 8-week companion group; *CG* control group

**Table 3:** Average attitudes of adolescents towards dementia and the elderly at different time points in experimental study

**Variable**	n	**Pre-test**	n	**Post-test**	n	**Post-test 1**^**st**^** month**	n	**Post-test 3**^**rd**^** month**	n	**Post-test 6**^**th**^** month**
		Mean (SD)		Mean (SD)		Mean (SD)		Mean (SD)		Mean (SD)
**Attitudes towards dementia**										
EGIV	42	94.76(13.73)	42	102.02(11.92)	41	102.10(12.57)	39	103.46(12.95)	39	103.08(12.77)
EGIII	40	86.55(12.85)	40	93.68(16.21)	36	99.19(17.50)	36	100.94(12.90)	29	99.69(13.27)
EGII	41	91.27(12.94)	41	96.68(12.37)	30	100.17(12.42)	26	101.19(13.29)	26	97.62(13.49)
EGI	40	97.48(14.40)	40	103.30(14.03)	35	99.86(13.62)	35	100.09(14.33)	35	98.94(13.25)
CG	37	91.57(10.92)	37	91.95(12.04)	35	94.20(13.83)	33	94.55(13.17)	31	94.13(11.72)
**Attitudes towards the elderly**^a^										
EGIV	42	63.05(19.60)	42	57.00 (17.08)	41	60.63 (19.34)	39	60.31(17.71)	39	60.54(17.06)
EGIII	40	72.38(14.67)	40	68.48 (17.63)	36	62.06 (19.71)	36	62.05(15.83)	29	65.93(12.65)
EGII	41	66.71(17.74)	41	58.63 (16.04)	30	57.07 (17.30)	26	57.08(17.51)	26	59.54(16.75)
EGI	40	62.85(17.93)	40	55.90 (17.64)	35	62.11 (16.91)	35	61.29(13.36)	35	62.69(16.12)
CG	37	57.86(20.38)	37	60.97 (15.82)	35	61.14 (15.90)	33	63.15(14.87)	31	66.26(14.76)

^a^a lower score indicated the better attitudes towards the elderly; EGI = 5-week exergaming group; EGII = 5-week companion group; EGIII = 8-week exergaming group; EGIV = 8-week companion group; *CG* control group

Table 4 shows that the time effect in attitudes toward dementia, at post-test, and at the 1-, 3- and post 6-month follow-ups were higher than the pretest, but not significant, at 0.378, 2.356, 2.762 and 1.473, respectively. After controlling for age, school grade, gender and participation in volunteer services for the elderly, the interaction effect of group by time in the 8-week exergaming group showed a significantly improved attitude toward dementia at post-test, and at the 1-, 3-, and 6-month follow-ups (95% confidence interval (CI) 2.585 to 10.908, *p* = 0.001; 95% CI 3.559 to 17.365, *p* = 0.003; 95% CI 5.000 to 18.612, *p* = 0.001; 95% CI 5.121 to 18.210, *p* < 0.001, respectively). Similarly, the attitude toward dementia in the 8-week companion group was significantly better than that of the control group both at the post-test and at the 6-month follow-up (95% CI 1.693 to 12.074, *p* = 0.009; 95% CI 0.263 to 13.023, *p* = 0.041, respectively). For the 5-week exergaming group, the attitude toward dementia showed no statistically significant difference compared with the control group at post-test, and at the 1-, 3-, and 6-month follow-ups. However, the attitude toward dementia in the 5-week companion group was significantly better than that in the control group only at post-test (95% CI 0.416 to 9.657, *p* = 0.033).

**Table 4:** Results for attitudes of adolescents towards dementia and the elderly at different time points

	**Attitudes towards dementia**			**Attitudes towards the elderly **^**a**^		
	*β*	95%CI	P	*β*	95%CI	P
Groups						
EGIV	2.257	-3.649–8.164	.454	6.797	-2.496–16.089	.152
EGIII	-6.783	-11.794- -1.771	.008**	15.072	6.753–23.390	< .001***
EGII	0.416	-4.835–5.667	.877	8.706	.297–17.115	.042
EGI	4.542	-1.258–10.342	.125	8.111	-.446–16.668	.063
CG (ref.)						
Times						
Post-test 6^th^ month	1.473	-2.457–5.402	.463	8.154	2.264–14.044	.007**
Post-test 3^rd^ month	2.762	-1.929–7.452	.249	6.137	-.157–12.430	.056
Post-test 1^st^ month	2.356	-1.430–6.141	.223	3.293	-3.424–10.009	.337
Post-test	0.378	-2.449–3.206	.793	3.108	-3.185–9.402	.333
Pre-test (ref.)						
Groups $\times$ Times						
EGIV $\times$ Post-test 6^th^ month	6.643	.263–13.023	.041*	-10.660	-19.309–2.011	.016*
EGIV $\times$ Post-test 3^rd^ month	5.739	-1.695–13.172	.130	-8.873	-17.314–.433	.039*
EGIV $\times$ Post-test 1^st^ month	4.716	-1.050–10.481	.109	-5.445	-15.136–4.246	.271
EGIV $\times$ Post-test	6.884	1.693–12.074	.009**	-9.156	-17.399–.913	.029*
EGIII $\times$ Post-test 6^th^ month	11.665	5.121–18.210	< .001***	-15.769	-23.453–8.084	< .001***
EGIII $\times$ Post-test 3^rd^ month	11.806	5.000–18.612	.001**	-16.889	-25.383–8.394	< .001***
EGIII $\times$ Post-test 1^st^ month	10.462	3.559–17.365	.003**	-14.406	-23.623–5.188	.002**
EGIII $\times$ Post-test	6.747	2.585–10.908	.001**	-7.008	-14.814-.798	.078
EGII $\times$ Post-test 6^th^ month	3.063	-2.908–9.033	.315	-11.358	-19.142–3.574	.004**
EGII $\times$ Post-test 3^rd^ month	5.351	-.661–11.363	.081	-11.802	-19.752–3.852	.004**
EGII $\times$ Post-test 1^st^ month	4.210	-1.233–9.652	.129	-9.270	-17.968–.572	.037*
EGII $\times$ Post-test	5.036	.416–9.657	.033*	-11.181	-19.077–3.285	.006**
EGI $\times$ Post-test 6^th^ month	-.317	-6.429–5.796	.919	-8.053	-16.288-.183	.055
EGI $\times$ Post-test 3^rd^ month	-.463	-7.311–6.386	.895	-7.435	-15.680-.809	.077
EGI $\times$ Post-test 1^st^ month	-.286	-6.567–5.996	.929	-3.763	-12.391–4.866	.393
EGI $\times$ Post-test	5.447	-.624–11.518	.079	-10.058	-19.085–1.031	.029*
Gender (ref. = female)	-2.660	-6.290-.970	.151	2.585	-1.985–7.154	.268
Age	4.225	2.040–6.409	< .001***	-2.026	-4.907-.854	.168
School grade	-13.440	-20.351–6.528	< .001***	8.434	-.556–17.9425	.066
Volunteer services^b^ (ref. = no)	2.494	.358–5.346	.087	-4.558	-8.681–.435	.030*

*CI* confidence interval, *Ref* reference group

^a^a lower score indicated the better attitudes towards the elderly;

^b^participation in volunteer services for the elderly; EGI = 5-week exergaming group; EGII = 5-week companion, group; EGIII = 8-week exergaming group; EGIV = 8-week companion group; *CG* control group

^*^ *p* < .05, ** *p* < .01, ***p < .001

In terms of the time effect in attitudes toward the elderly, the post-test, 1-, 3- and 6-month measurements were higher than the pretest, at 3.108, 3.293, 6.137, and 8.154, respectively; the post 6-month measurement was significant. The interaction effect of group by time in the 8-week exergaming group showed a better attitude toward the older adults than the control group at the 1-, 3-, and 6-month follow-ups (95% CI -23.623 to -5.188, *p* = 0.002; 95% CI -25.383 to -8.394, *p* < 0.001; 95% CI -23.453 to -8.084, *p* < 0.001, respectively). The 8-week companion group showed a better attitude toward the older adults than the control group at the post-test and at the 3- and 6-month follow-ups (95% CI -17.399 to -0.913, *p* = 0.029; 95% CI -17.314 to -0.433, *p* = 0.039; 95% CI -19.309 to -2.011, *p* = 0.016, respectively).

However, the attitude of the 5-week exergaming group toward the older adults was significantly better than that of the control group only at post-test (95% CI -19.085 to -1.031, *p* = 0.029). The 5-week companion group showed a better attitude toward the older adults than the control group at post-test, and at the 1-, 3-, and 6-month follow-ups (95% CI -19.077 to -3.285, *p* = 0.006; 95% CI -17.968 to -0.572, *p* = 0.037; 95% CI -19.752 to -3.852, *p* = 0.004; 95% CI -19.142 to -3.574, *p* = 0.004, respectively) (Table 4). Overall, the present results show that the 8-week intervention is more effective than the 5-week intervention in improving the attitudes of adolescents toward dementia. Finally, the attitude toward the older adults of the 8-week companion group was significantly better than that of the control group both at post-test and at the 6-month follow-up. The attitude toward the older adults of the 5-week companion group was significantly better than that of the control group at the post-test and at the 1-, 3- and 6-month follow-ups.

## Discussion

As shown in Table 2, there were significantly more females than males in all groups. This result was consistent with the nationwide survey. In Taiwan, the sex ratio of adolescents (12–17 years old) from 2018-2020 was 1.09 (male: female), but the sex ratio (female: male) of participating volunteer service was 1.49 (Statistics from Department of Gender Equality, Executive Yuan in Taiwan [33]. The higher ratio is most likely due to the nearly global tendency of nursing and care facility staff being dominated by females, and thus the adolescent volunteer ratio for this study more likely reflects the realistic social interest.

The 8-week exergaming group showed a significantly better attitude toward dementia than the control group at the 6-month follow-up. Interestingly, the 8-week exergaming group showed the lowest initial attitude scores toward dementia among the five groups. Thus, the results suggest that the act of providing regular and extended support and assistance to older adults with dementia in playing exergames caused the adolescents to become more familiar with the characteristics and needs of their matched older adults and to improve their attitudes accordingly. The positive outcome in the present study was mainly the result of applying the principle of gameplay from easy to complicated/cooperative. Starting with the bowling game, older adults followed the adolescents’ instructions, watched their moves and learned how to play. From the above learning process, the relationship between the adolescents and the older adults was established. As time passed, they advanced to the more complicated games, e.g., fruit ninja and target shoot, which required that both the adolescent and older adult played as a team cooperatively. Finally, two adolescent and older adult dyads dual play [34] increased their tacit agreement through body movement, verbal reminders, and competition.

Second, as described in the study of Dove and Astell [35], older adults with dementia tend to forget the steps in a game, and thus, the adolescents are required to use learned tasks, breakdown, verbal cues and gesture demonstrations to help the older adults remember and re-engage with the game. Third, adolescents, as trainers, have learned to observe the elders’ needs and coach the program but not engage in “overhelping”. This type of cooperation would better establish a level of trust between the adolescents and the older adults. The present results suggest that these interactions are beneficial to improving adolescents’ attitudes toward older adults with dementia. Furthermore, after becoming aware of the cognitive and physiological limitations of their matched older adult and observing the performance improvement made by them in response to their interventions, it seems that the adolescents felt a sense of accomplishment, which then translated into an improved long-term attitude toward the older adult with dementia. It is noted that the present results are consistent with those of Zhang et al. [36], which showed the benefits of cooperative exergames in improving the attitudes of college-age students toward older adults.

The 5-week exergaming group showed a significantly improved attitude toward dementia compared with the control group only at the posttest. This finding suggests that a 5-week exergaming intervention is insufficient to produce a long-term change in adolescents’ attitudes toward dementia. Mainly, some of the older adults with dementia were unfamiliar with the games and required particularly strong encouragement from the adolescents or their peers to participate in the assigned games. This finding is consistent with a previous study. A brief period of contact did not change adolescents’ attitudes toward older adults [18].

Both the 8-week companion group and the 5-week companion group showed an improved attitude toward older adults following the intervention. Moreover, the effect appeared to be long lasting. This finding is similar to that of a meta-analysis by Burnes et al. [37] and is also consistent with previous studies that showed that intergenerational arts programs are effective in improving the attitudes of adolescents toward dementia [38, 39]. However, even though the 8-week and 5-week companion groups both showed better attitudes toward dementia than the control group at post-test, only the 8-week companion group showed a statistically significant improvement in attitude at the 6-month follow-up. This finding suggests that the process of companionship of older adults with dementia during the performance of simple activities requires less cooperation and brain stimulus than exergaming, and, thus, shorter companionship programs (e.g., 5 weeks) are less effective in promoting long-term attitudinal change among adolescents than longer programs. The results further suggest that in the event of shorter interventions, follow-up interventions are required to sustain the attitudinal shift of adolescents over the longer term. Moreover, for 8-week interventions, annual follow-up tests should be performed to ascertain the permanency (or otherwise) of the attitudinal shift, with further interventions implemented if needed.

Generally, adolescents tend to have little contact with older adults with dementia outside of their own families. Therefore, older adults with mental illness are at particular risk of being shunned [40]. According to Sanders et al. [31], adolescents commonly regard older adults as *unattractive, complaining, and conservative*. This negative impression of older adults is incorporated into the Attitudes Toward Elderly scale developed by Hawkins [30] in the *sick*, *inflexible*, *conservative*, *unattractive*, *intolerant*, *pessimistic* and *complaining* items. The study of Tan et al. [41] similarly showed that Chinese college students regard older adults as *inflexible* and *unattractive*. The present study showed that both the 8-week and 5-week groups regarded the older adults negatively as being “*inflexible*”, “*unattractive*”, and “*complaining*” in the pretest. However, following interaction through the exergaming or companionship programs, the attitudes toward older adults were reversed, becoming *kind*, *generous*, *friendly* and *good*, except for *inflexible,* which still existed over time.

### Limitations

To participate in intergenerational activities, both adolescents in the schools and older adults in daycare centers need to adjust their schedules. Matching schools and daycare centers is challenging and cannot be performed simply by random assignment. Thus, future studies should consider strategies to enlarge the sample size such that randomized allocation can be more easily applied.

## Conclusions

The results showed that both 8-week and 5-week exergaming or companionship programs can improve adolescents' attitudes toward older adults. The findings also support the hypothesis of contact theory that more time for contact could reduce prejudice. Furthermore, 8 weeks of exergaming significantly improve the attitudes of adolescents toward both dementia and older adults. Thus, regular or long-term collaboration between schools and daycare centers through exergaming appears to be an effective means of increasing the contact time of adolescents with older adults and improving their understanding and attitudes accordingly. For the case where concern regarding exergaming games prompts an adverse emotional response in older adults with dementia, regular companionship programs, in which adolescents simply accompany older adults while performing simple activities, may provide an effective alternative approach.

## Data Availability

The data supporting the findings of this study are available within the article. The data during the current study are not publicly available due ethical restrictions. The datasets are available upon reasonable request from the corresponding author and first author.

## Abbreviations

ANOVA: Analysis of variance
CI: Confidence interval

## References

[1] World Health Organization. Dementia. 2021. https://www.who.int/news-room/fact-sheets/detail/dementia Accessed 3 Mar 2022.

[2] North MS, Fiske ST (2012). An inconvenienced youth? Ageism and its potential intergenerational roots. Psychol Bull.

[3] Mulvey KL, Hitti A, Killen M (2010). The development of stereotyping and exclusion. Wiley Interdisciplinary Rev: Cognitive Sci.

[4] Farina N, Hughes LJ, Griffiths AW, Parveen S (2020). Adolescents’ experiences and perceptions of dementia. Aging Mental Health.

[5] Alzheimer’s Disease International. The World Alzheimer Report 2019: Attitudes to dementia. https://www.alz.co.uk/research/WorldAlzheimerReport2019.pdf Accessed 19 Jul 2020.

[6] Fuh JL, Wang SJ, Juang KD (2005). Understanding of senile dementia by children and adolescents: Why grandma can't remember me?. Acta Neurol Taiwan.

[7] Venters S, Jones CJ (2021). The experiences of grandchildren who provide care for a grandparent with dementia: a systematic review. Dementia (London, England).

[8] Allen J, Oyebode JR, Allen J (2009). Having a father with young onset dementia: the impact on well-being of young people. Dementia.

[9] Bergman EJ, Erickson MA, Simons JN (2014). Attracting and training tomorrow's gerontologists: What drives student interest in aging?. Educ Gerontol.

[10] Gaggioli A, Morganti L, Bonfiglio S, Scaratti C, Cipresso P, Serino S (2014). Intergenerational group reminiscence: a potentially effective intervention to enhance elderly psychosocial wellbeing and to improve children's perception of aging. Educ Gerontol.

[11] Kim J, Lee J (2018). Intergenerational program for nursing home residents and adolescents in korea. J Gerontol Nurs.

[12] Kolodinsky J, Cranwell M, Rowe E. Bridging the generation gap across the digital divide: Teens teaching internet skills to senior citizens. J Ext. 2002;40(3). https://archives.joe.org/joe/2002june/rb2.php. Accepted 19 July 2021.

[13] Chua P-H, Jung Y, Lwin MO, Theng Y-L (2013). Let’s play together: Effects of video-game play on intergenerational perceptions among youth and elderly participants. Comput Human Behav.

[14] Thompson EH, Weaver AJ (2016). Making connections: The legacy of an intergenerational program. Gerontologist.

[15] Lytle A, Levy SR (2017). Reducing ageism: Education about aging and extended contact with older adults. Gerontologist.

[16] Lytle A, Macdonald J, Apriceno M, Levy SR. Reducing ageism with brief videos about aging education, ageism, and intergenerational contact. Gerontologist. 2021;61(7):1164–8. 10.1093/geront/gnaa167 33103201

[17] Celdrán M, Villar F, Triadó C (2014). Thinking about my grandparent: How dementia influences adolescent grandchildren's perceptions of their grandparents. J Aging Stud.

[18] Baker JR, Webster L, Lynn N, Rogers J, Belcher J (2017). Intergenerational Programs May Be Especially Engaging for Aged Care Residents With Cognitive Impairment: Findings From the Avondale Intergenerational Design Challenge. Am J Alzheimers Dis Other Demen.

[19] Pettigrew TF (1998). Intergroup contact theory. Annu Rev Psychol.

[20] Allport GW. The effect of contact. The nature of prejudice: Cambridge, Mass. : Addison-Wesley; 1954. p. 261–82.

[21] Oh Y, Yang S, editors. Defining exergames & exergaming. Meaningful Play 2010; 2010 October 21 - 23; Michigan State University, East Lansing, MI, USA.

[22] Vanden Abeele V, De Schutter B (2010). Designing intergenerational play via enactive interaction, competition and acceleration. Pers Ubiquitous Comput.

[23] Khoo ET, Merritt T, Cheok AD (2009). Designing physical and social intergenerational family entertainment. Interact Comput.

[24] Ruehrlinger M, Gattringer F, Stiglbauer B, Hagler J, Lankes M, Holzmann C, editors. It is not rocket science. It is collaborative play for old and young! 2018 IEEE 6th International Conference on Serious Games and Applications for Health (SeGAH); 2018 May 16–18.

[25] Rice M, Tan WP, Ong J, Yau LJ, Wan M, Ng J, editors. The dynamics of younger and older adult's paired behavior when playing an interactive silhouette game. CHI '13: CHI Conference on Human Factors in Computing Systems; 2013 April 27-May 2; Paris, France: Association for Computing Machinery.

[26] Kern D, Stringer M, Fitzpatrick G, Schmidt A, editors. Curball-A prototype tangible game for inter-generational play. 15th IEEE International Workshops on Enabling Technologies: Infrastructure for Collaborative Enterprises (WETICE'06); 2006 June 26–28

[27] Xu X, Li J, Tan Phat P, Salmon CT, Theng Y (2016). Improving psychosocial well-being of older adults through exergaming: the moderation effects of intergenerational communication and age cohorts. Games Health J.

[28] Tsolaki M, Makri M (2020). “Bridge” project: An intergenerational approach with prototype games for young people and people with dementia. Alzheimers Dement.

[29] O'Connor M, McFadden S (2010). Development and psychometric validation of the dementia attitudes scale. J Alzheimers Dis.

[30] Hawkins MJ (1996). College students' attitudes toward elderly persons. Educ Gerontol.

[31] Sanders GF, Montgomery JE, Pittman JF, Balkwell C (1984). Youth's attitudes toward the elderly. J Appl Gerontol.

[32] Rothman KJ (1990). No Adjustments Are Needed for Multiple Comparisons. Epidemiology.

[33] Gender Equality Committee of the Executive Yuan ROC. Volunteer services statistics database. 2018. https://www.gender.ey.gov.tw/gecdb/Stat_Statistics_DetailData.aspx?sn=pLlshvcDKuKDTLCdrqBQOQ%40%40 Accessed 11 Mar 2022.

[34] Vanden Abeele V, De Schutter B (2010). Designing intergenerational play via enactive interaction, competition and acceleration. Personal Ubiquitous Comput.

[35] Dove E, Astell AJ (2019). The kinect project: Group motion-based gaming for people living with dementia. Dementia (London).

[36] Zhang F, Kaufman D, Schell R, Salgado G, Seah ETW, Jeremic J (2017). Situated learning through intergenerational play between older adults and undergraduates. Int J Educ Technol High Educ.

[37] Burnes D, Sheppard C, Henderson CR, Wassel M, Cope R, Barber C (2019). Interventions to reduce ageism against older adults: A systematic review and meta-analysis. Am J Public Health.

[38] Lokon E, Li Y, Parajuli J (2017). Using art in an intergenerational program to improve students' attitudes toward people with dementia. Gerontol Geriatr Educ.

[39] Yamashita T, Kinney JM, Lokon EJ (2013). The impact of a gerontology course and a service-learning program on college students' attitudes toward people with dementia. J Appl Gerontol.

[40] Katona C, Chiu E, Adelman S, Baloyannis S, Camus V, Firmino H (2009). World psychiatric association section of old age psychiatry consensus statement on ethics and capacity in older people with mental disorders. Int J Geriatr Psychiatry.

[41] Tan PP, Zhang N, Fan L (2004). Students' attitudes toward the elderly in the people's republic of china. Educ Gerontol.

